# Volumetric imaging reveals VEGF-C-dependent formation of hepatic lymph vessels in mice

**DOI:** 10.3389/fcell.2022.949896

**Published:** 2022-08-16

**Authors:** Stefanie Bobe, Daniel Beckmann, Dorothee Maria Klump, Cathrin Dierkes, Nils Kirschnick, Esther Redder, Nadine Bauer, Michael Schäfers, Raghu Erapaneedi, Benjamin Risse, Serge A. van de Pavert, Friedemann Kiefer

**Affiliations:** ^1^ European Institute for Molecular Imaging, University of Münster, Münster, Germany; ^2^ Gerhard-Domagk-Institute of Pathology, University Hospital Münster, Münster, Germany; ^3^ Institute for Geoinformatics, University of Münster, Münster, Germany; ^4^ Centre National de la Recherche Scientifique (CNRS), National Institute for Health and Medical Research (INSERM), Centre d'Immunologie de Marseille-Luminy (CIML), Aix-Marseille University, Marseille, France

**Keywords:** liver, VEGF-C, portal tracts, lymphatic vessel, lymphatic endothelial cells, whole mount imaging, 3D reconstruction, portal vein

## Abstract

The liver is a major biosynthetic and detoxifying organ in vertebrates, but also generates 25%–50% of the lymph passing through the thoracic duct and is thereby the organ with the highest contribution to lymph flow. In contrast to its metabolic function, the role of the liver for lymph generation and composition is presently severely understudied. We took a rigorous, volume imaging-based approach to describe the microarchitecture and spatial composition of the hepatic lymphatic vasculature with cellular resolution in whole mount immune stained specimen ranging from thick sections up to entire mouse liver lobes. Here, we describe that in healthy adult livers, lymphatic vessels were exclusively located within the portal tracts, where they formed a unique, highly ramified tree. Ragged, spiky initials enmeshed the portal veins along their entire length and communicated with long lymphatic vessels that followed the path of the portal vein in close association with bile ducts. Together these lymphatic vessels formed a uniquely shaped vascular bed with a delicate architecture highly adapted to the histological structure of the liver. Unexpectedly, with the exception of short collector stretches at the porta hepatis, which we identified as exit point of the liver lymph vessels, the entire hepatic lymph vessel system was comprised of capillary lymphatic endothelial cells only. Functional experiments confirmed the space of Disse as the origin of the hepatic lymph and flow *via* the space of Mall to the portal lymph capillaries. After entry into the lymphatic initials, the lymph drained retrograde to the portal blood flow towards the exit at the liver hilum. Perinatally, the liver undergoes complex changes transforming from the main hematopoietic to the largest metabolic organ. We investigated the time course of lymphatic vessel development and identified the hepatic lymphatics to emerge postnatally in a process that relies on input from the VEGF-C/VERGFR-3 growth factor—receptor pair for formation of the fully articulate hepatic lymph vessel bed.

## Introduction

The lymphatic vascular system ensures tissue homeostasis, chylomicron transport, and immune cell trafficking. Lymph capillaries start as blind-ending structures draining *via* collecting vessels and interposed lymph nodes. The lymph vasculature is connected to the blood vasculature by the thoracic and the right lymphatic duct. While capillary lymphatic vessels are specialized for uptake of fluid and immune cells, collectors are designated to lymph transport (Aspelund et al., 2016). In mice, the first primordial lymphatic structures appear at days 10.5–11.5 of embryonic development (E10.5-E11.5) (Srinivasan et al., 2007) and their formation highly depends on the transcription factor Prospero homeobox 1 (PROX-1) (Wigle et al., 2002) and signaling via the VEGF-C/VEGFR-3 ligand-receptor pair (Hägerling et al., 2013).

The hepatic vasculature is characterized by a complex architecture where the hepatic artery and portal vein form a double blood supply delivering oxygen-rich arterial and venous blood from the spleen and gastrointestinal tract. Accompanied by branches of the bile duct, both vessels run in the portal tracts of the liver and merge into the hepatic sinusoids, where plasma gains access to the metabolic activity of hepatocytes. Central veins collect the blood from sinusoids and guide it to the Vena cava. Alongside the sinusoids, the liver parenchyma is organized into three metabolic zones, covering the periportal (zone 1), pericentral (zone 3) and intermediary area (zone 2).

About 25%–50% of the lymph in the thoracic duct originates from the liver (Mobley et al., 1989; Ohtani and Ohtani, 2008) and the hepatic lymph is characterized by an especially protein-rich composition (Tso et al., 1983). Rouvière and Comparini described drainage of lymph from the space of Disse *via* three pathways; the first running alongside the portal vessels to the porta, the second following the central vein and the third reaching and following the hepatic capsule (Comparini, 1969). Recently, the drainage pattern of hepatic lymph was shown to follow the segmental structure and involve the lymph nodes of the hepatoduodenal ligament and the mediastinal thoracic lymph node (Frenkel et al., 2020).

Hepatic lymph vessels are not only important for physiological tissue homeostasis, but also play an important role during pathological conditions (Jeong et al., 2022). An increase in hepatic lymphatic vasculature was described for idiopathic portal hypertension (Oikawa et al., 1998) as well as liver fibrosis and cirrhosis (Yamauchi and Michitaka, 1998; Tamburini et al., 2019). Hepatocellular carcinoma (HCC) is associated with lymphangiogenesis and expression of the vascular endothelial growth factor C (VEGF-C) correlates with size and metastasis formation of HCC (Yamaguchi et al., 2006).

Detailed knowledge on the spatial arrangement of the vascular systems is paramount for their functional understanding. Although the last years saw substantial progress in the investigation of lymphatic development and morphology, a detailed and comprehensive three-dimensional (3D) description of the hepatic lymph vessels in mice and the details of their development are still missing. The dense and pigmented hepatic tissue challenges volumetric optical imaging and molecular lymphatic markers lack specificity in the liver.

Here, we used light sheet and confocal microscopy-based volume imaging to visualize the lymphatic vasculature in specimens ranging from thick sections to complete liver lobes. We identified the VEGF-C/VEGFR-3 axis as a crucial player in the formation of the hepatic lymphatic vascular network.

## Material and methods

### Mouse strains

Wild-type, Vegfr-3^+/LacZ^ mice (Dumont et al., 1998) of C57Bl/6 genetic background and VEGF-C^+/LacZ^ mice of CD1 background (Karkkainen et al., 2004) were analyzed. Wild-type embryos and pups were examined at different developmental stages between E12.5 and P21. Embryonic staging was determined by the day of the vaginal plug (E0.5). Adult animals were analyzed at the age of 10–20 weeks. All animal husbandry and experimentation was performed according European law as specified in the directive EU RL 2010/63 EEC of the Council on the approximation of laws, regulations and administrative provisions of the member states and approved by the responsible local ethics committees.

### Ultramicroscopy

Immunostained and optically cleared liver samples were imaged on a LaVision Ultramicroscope (LaVision BioTec, Bielefeld) with a step size of 2 µm. 3D reconstruction was performed on high end commodity hardware using the volume rendering software package Voreen (voreen.uni-muenster.de) (Meyer-Spradow et al., 2009; Hägerling et al., 2017)

### Immunofluorescence whole mount staining

Liver specimens were permeabilized using 5% Triton-X 100/PBS at 37°C and blocked using Permblock solution (3% bovine serum albumine, 0.5% Tween^®^20, in PBS). Immunostaining was performed freely floating in antibody solution at 37°C. After each staining step the specimens were extensively washed in PBS-T (0.1% Tween^®^20 in PBS).

### Immunofluorescence staining of sections

Livers were fixed for 2–4 h in 4% formaldehyde/PBS and washed in PBS. Vibratome sections of 100–300 µm thickness were permeabilized using 0.5% Triton-X 100/PBS and blocked using Permblock solution. Immunostaining was performed freely floating in antibody solution. After each staining step the sections were extensively washed in PBS-T. After mounting in Mowiol (Calbiochem, United States), confocal images were captured with a Zeiss LSM880 confocal microscope (10x, NA = 0.45; 20x, NA = 0.8; 63x, NA = 1.2).

### Optical clearing

Liver specimens were optically cleared in BABB (Murray’s solution consisting of a 1:2 ratio of benzyl alcohol and benzyl benzoate). After whole mount immunostaining, the specimens were embedded in low melting point agarose and dehydrated using escalating methanol concentrations (50%, 70%, 95%, and 99.8%). Methanol was then exchanged by a 1:1 mixture of methanol and BABB and finally replaced by BABB.

### Portal vein injection

Mice were anaesthetized by intraperitoneal injection of Ketamine (125 mg/kg body weight) and Xylazine (12.5 mg/kg body weight). Surgical depth of the anesthesia was reached after a few minutes, indicated by the absence of the paw withdrawal reflex. The abdomen was opened and the intestine was moved to the left side allowing free access to the portal vein. The portal vein was cannulated with a 27 G ¾ needle and the antibody solution (0.75 mg/kg body weight) was injected. After 3 min the thoracic cavity was opened and the lung was cut. The liver was then perfused with heparin/PBS (10 U/ml) and 4% PFA *via* the portal vein using a peristaltic pump and afterwards explanted from the mouse.

### Antibodies

The following antibodies were used: goat anti-LYVE-1 (polyclonal, R&D, AF2089), rat anti-PECAM-1 (clone 5D2.6 and IG5.1, provided by D. Vestweber, Max Planck Institute for Molecular Biomedicine Munster), rabbit anti-LYVE-1 (polyclonal, Reliatech), goat anti-LDLR (polyclonal, R&D, AF2255), rabbit anti-GS (polyclonal, Sigma-Aldrich, G2781), rabbit anti-CK19 (polyclonal, proteintech, 14965-1-AP), mouse anti-smooth muscle actin (SMA)-Cy3 (clone 1A4, Sigma), rat anti-Lyve-AlexaFluor594 (clone 223322, R&D), secondary antibodies coupled to AlexaFluor488, 568 and 647 (Invitrogen).

### Quantitative analysis of the lymphatic network

The acquired image volumes were converted into the easy-to-use HDF5 format and the relevant inner volume was extracted by using a global erosion operation on the slice-wise concave hull of the imaged liver tissue sample. For segmentation, straightforward hysteresis thresholding was utilized, of which the upper and lower limit were set manually for each volume. Subsequently, connected component analysis and size thresholding was used to remove small and erroneous artifacts. To ensure segmentations of lymphatic vessels only, all resultant volumes were manually inspected and incorrectly extracted blood vessels were removed by an expert. The resultant 3D segmentation volume was further processed using a graph and feature extraction pipeline (VIPAR) adapted from Hägerling et al. (2017) and Drees et al. (2021) to obtain the skeletonized volume and the underlying graph structure after iterative refinement.

## Results

### Identifying hepatic lymphatic vessels

Several marker proteins are well established for the identification of lymph vessels, including the transcription factor PROX-1, the transmembrane tyrosine kinase receptor VEGFR-3, the lymphatic vessel endothelial hyaluronan receptor 1 (LYVE-1) and the surface glycoprotein podoplanin (PDPN) [summarized in (Dierkes et al., 2018)]. While these proteins are differentially expressed on blood and lymphatic endothelial cells (LECs), however, none is exclusive for LECs. This issue is particularly prevalent in the murine liver, where the LEC markers are also prominently expressed on non-endothelial cells. In order to develop a staining strategy for reliable identification of lymphatic vessels, we analyzed the tissue distribution of these candidate LEC markers and established co-staining protocols for the pan-endothelial marker PECAM-1, the sinusoidal EC marker LDLR, the zone 3-enzyme glutamine synthase (GS) and the biliary protein cytokeratin 19 (CK19).

**FIGURE 1:**
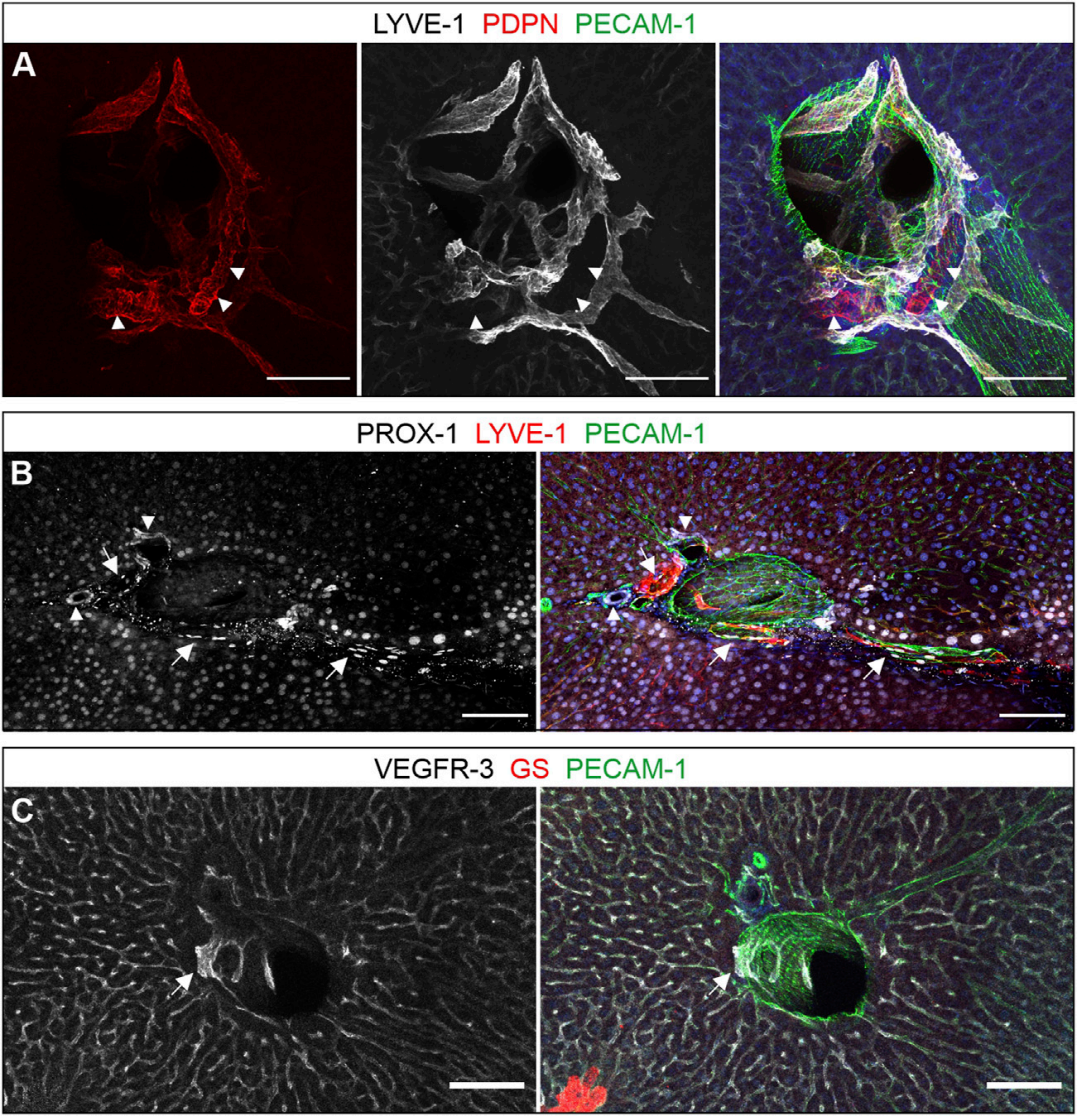
Lymphatic surface markers in the liver. (**A–C)**: Vibratome sections (300 µm) immunostained for the proteins indicated above the panels are shown as maximum intensity projections. **(A)**: PDPN and LYVE-1 co-localized on lymphatic vessels, while exclusive PDPN expression was restricted to bile ducts (arrowheads). **(B)**: Prox1 was detected on LYVE-1-positive lymphatics (arrows), bile ducts (arrowheads) and surrounding hepatocytes. **(C)**: VEGFR-3 was expressed on sinusoids as well as lymphatic vessels (arrow), here next to a portal venous branch as indicated by the absence of GS staining on neighboring hepatocytes. **(A–C)**: Scale bars = 100 µm, nuclear counterstaining with Hoechst.

**FIGURE 2:**
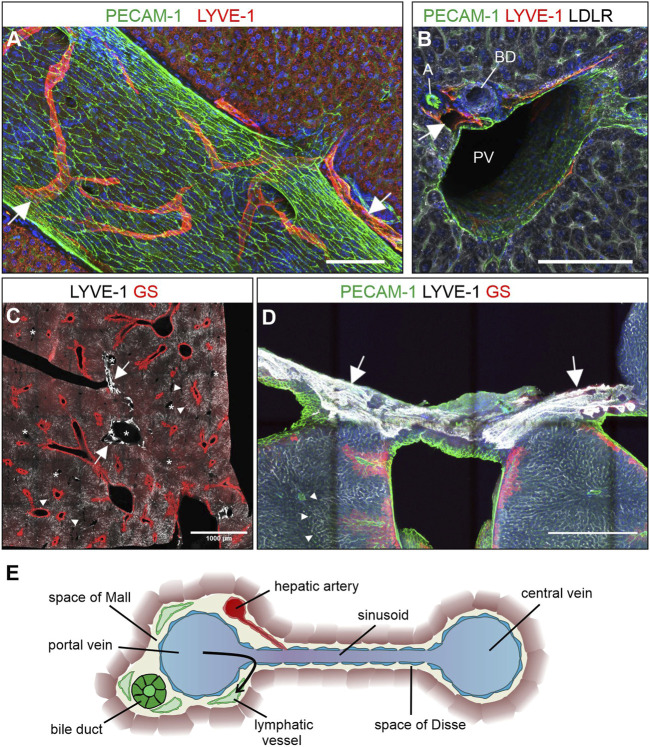
Lymphatic vessels are found in portal tracts. (**A–E)**: Vibratome sections (300 µm) were stained for the proteins indicated above and maximum intensity projections are shown. **(A)** Lymphatic vessels identified by LYVE-1 staining ran alongside and perpendicular to large portal venous vessels. Scale bar = 100 µm. **(B)** Cross-section of a portal tract showing lymphatic vessels (arrow) next to branches of the portal vein (PV), the hepatic artery **(A)**, and the bile duct (BD). Scale bar = 100 µm. **(C)** Sinusoidal LYVE-1-staining was most pronounced on the intermediate zone 2 sinusoids representatively indicated by arrowheads. Zone 3 sinusoids were identified by their proximity to the GS-positive hepatocytes flanking the central veins. The comparatively low LYVE-1 staining on zone 1 sinusoids facilitated identification of strongly LYVE-1-positive lymphatics (arrows) near the portal tracts (representative annotation with asterisks). Scale bar = 1000 µm. **(D)**: LYVE-1 positive vessels (arrows) were found near the capsule around hepatic veins identified by PECAM-1 and GS staining. Arrowheads indicate representative zone 2 sinusoids. Scale bar = 500 µm. (**A,B,D)** Nuclear counterstaining with Hoechst. (**E)**: Schematic cross-section through a peripheral portal tract, sinusoid, and central vein. Besides branches of the bile duct, portal vein, and hepatic artery, also lymphatic vessels were a regular part of the portal tract.

Although LYVE-1 is expressed on sinusoids, good differentiation of lymphatic structures in the proximity of larger vessels was still possible as LYVE-1 expression was most pronounced in zone 2 sinusoidal endothelial cells, but weaker in zone 1 and 3 (Figures 2C,D, arrowheads). Furthermore, the signal detected on lymphatic vessels was clearly higher than on sinusoids. PDPN largely co-localized with staining for LYVE-1, verifying its presence on hepatic lymph vessels. However, PDPN was also found prominently expressed on bile ducts (Figure 1A). Besides lymphatic vessels, also hepatocytes are known to stain positive for nuclear PROX-1 (Figure 1B) which we readily verified. While the characteristic oblong shape of EC nuclei can be used for further distinction, we valued this parameter less reliable. While VEGFR-3 was found on lymphatic vascular structures, its predominant appearance was a homogenous staining of all sinusoids (Figure 1C).

Based on the pronounced staining of PROX-1, VEGFR3 and PDPN on non-lymphatic structures, we elected to focus on LYVE-1 as lymphatic marker. However, since LYVE-1 expression is generally restricted to lymphatic capillaries, probing of the other markers was still required to identify lymphatic collectors.

### Lymphatic vessels of the liver were located in portal tracts

Hepatic lymph vessels have been detected in the portal tracts, subcapsular and alongside the central veins (Trutmann and Sasse, 1994a; Tanaka and Iwakiri, 2016). We revisited the question of the spatial architecture of liver lymph vessels by confocal and light sheet microscopy of whole mount stained thick sections and entire liver lobes of adult healthy mice. Applying the validated marker combinations, we found LYVE-1-positive lymphatic vessels exclusively located in close proximity to portal vessels (Figures 2A,B), while we did not detect lymph vessels near central veins, which were identified by GS staining (Figure 2C).

LYVE-1-positive vascular structures were also found in sections close to the hepatic venous confluence in the median lobe, an anatomical position that made it unlikely that they arose from portal lymph vessels (Figure 2D). The consistently noted absence of lymphatic vessels from the proximity of the central veins in all our immunofluorescence stainings and their appearance at the hepatic surface perpendicular to the hepatic veins suggested that these vessels were superficially associated rather than originating from the liver.

The observation of lymphatic vessels exclusively near portal tracts was confirmed in whole mount stainings of the complete caudate lobe for VEGFR-3. Despite the VEGFR-3-positive sinusoids, lymphatic vessels were successfully identified by following vessel branches, which ultimately derived from the proximity of the portal vein. (Supplementary Video S1, Figure 2E).

### Volumetric imaging of lymphatic hepatic vessels

**FIGURE 3:**
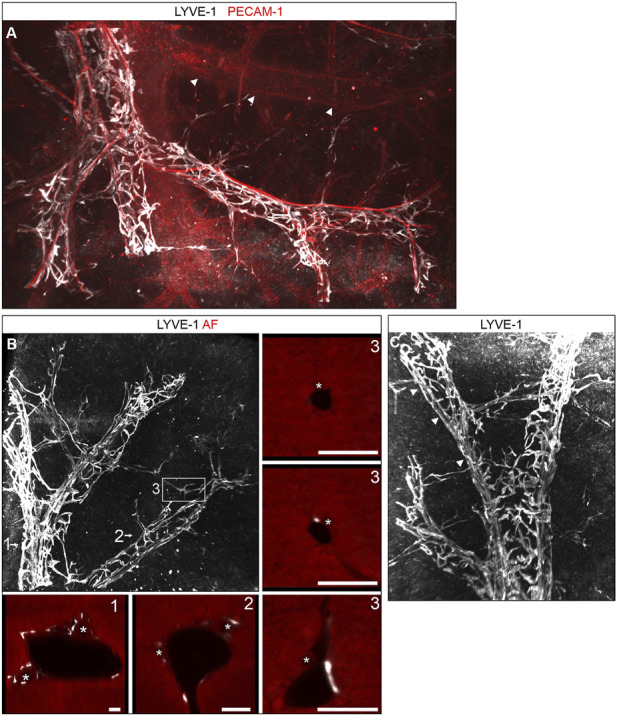
3D morphology of hepatic lymphatic vessels. (**A)**: Digital 3D reconstruction of hepatic lobes whole mount immunostained for LYVE-1 and PECAM-1. The portal vein branches were covered by a dense network of lymph vessels. A central vein in the background (arrowheads) lacked lymphatic vessels. The lymphatic network consisted of spiky initials enwrapping the portal vein perpendicular to its orientation and of straight vessels running alongside the portal arteries (intense PECAM-1 staining). **(B,C)** Digital 3D reconstruction of liver specimens whole mount immunostained for LYVE-1, revealed a dense lymphatic network. Note the straight LYVE-1 positive vessels (arrowheads). (**B)** (numbers side panels): Single optical sectional planes from the regions indicated by the numbers in the central panel. Besides LYVE-1 the autofluorescence (AF) signal at 546 nm (red) is shown for anatomical orientation. Lymphatics surrounded portal vein branches and bile ducts (asterisks) in larger portal tracts (1, 2). Initial bile ducts were not yet associated with lymph vessels, however the smallest lymphatic vessels appeared next to small bile ducts. At portal vein branch points this connection was transiently lost (3). Scale bar = 100 µm.

While the confocal analysis of thick vibratome sections allowed a detailed study of lymphatic morphology with subcellular resolution, the mesoscopic light sheet microscopy enabled us to visualize the lymphatic network on a larger scale in the context of whole hepatic lobes or the entire liver (Supplementary Video S2). Whole mount staining confirmed and extended our observations obtained from vibratome sections. An extensive network of blind ending lymphatic vessels covered the branches of the portal vein (Figures 3A–C). Different from large lumen, smooth dermal lymphatic initials, the initials of the hepatic lymph vessel tree were composed of spiky finger-like protrusions often clasping the portal tracts akin the rungs of a ladder. Connected to these protrusions, straight lymph vessels were located running alongside and surrounding the larger bile ducts (Figure 2B). Figure 3B shows the localization of LYVE-1-stained lymphatics and bile ductules discernible by their autofluorescence at 546 nm. In the periphery of the lymphatic and bile ductular tree, small bile ducts first appeared without associated lymphatic vessel, which subsequently as smallest lymph vessels joined the bile ducts peripherally and accompanied them centrally towards the liver hilum. Once this dyad had formed, bile duct and lymphatic vessel were found associated except for branching points of the portal vein, where both transiently dissociated. The mesoscopic large volume analysis provided no evidence for lymph vessels near central veins, which were readily identified by the absence of accompanying arterial and bile duct branches (Figure 3A, arrowheads).

### Lymph flow in the murine liver

**FIGURE 4:**
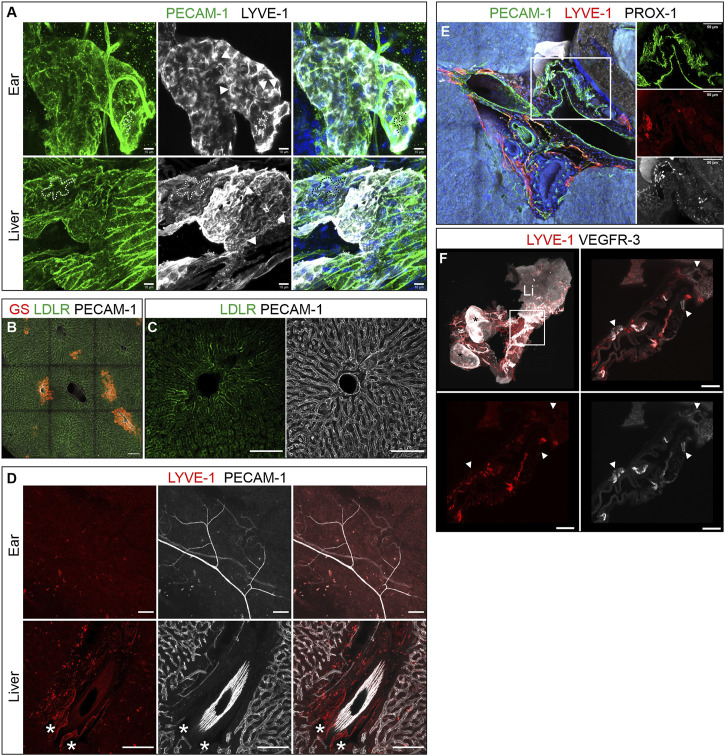
Lymph flow in the murine liver. **(A)** Hepatic lymph vessels were composed of capillary lymphatic endothelial cells (LECs). Like LYVE-1 positive dermal lymph capillaries (top row), hepatic LECs displayed the prototypic oak-leave shape of capillary (cap) LECs and formed blind ending spikes with a highly irregular wide lumen. Dashed lines outline the cell border of one exemplary LEC in each sample. Arrowheads indicate LYVE-1 positive primary valves of capLECs. Scale bar = 10 µm. **(B–D)**: Hepatic blood and lymph flow tracking *via* PVI of anti-PECAM-1, anti-LDLR and/or anti-LYVE-1 antibodies. Scale bar = 100 µm. **(B)**: GS immunostaining identified zone 3 sinusoids and central veins. **(C)** LDLR immune reactivity was limited to zone 1-sinusoids (absence of GS-staining), while PECAM-1 staining was homogenous **(D)**: Asterisks indicate LYVE-1-positive lymphatics in the liver after PVI, while no lymphatic vessels were identified in a whole mount preparation of the ear. **(E)**: Immunostaining (vibratome section of the right lobe, 300 µm) of a large portal tract near the porta. LYVE-1-neative, but PROX-1-positive collecting lymph vessels were identified in the region surrounded by the rectangle (details in panels on the right side). Scale bar = 50 µm. **(F)**: 3D reconstruction of a whole mount stained portal vein connected to the liver (Li) and two attached lymph nodes (asterisks). Note the lymph vessels wrapping around the large vein. Magnifications of the boxed area (white rectangle, top left panel) identified VEGFR-3-positive, LYVE-1-negative collecting vessels. A/E: Hoechst counterstaining, blue.

LYVE-1 expression, oak-leaved shaped lymphatic endothelial cells and button junctions are hallmarks of lymphatic capillaries (Petrova und Koh, 2020). The widespread LYVE-1 expression of virtually the entire intrahepatic lymph vessel tree appeared to indicate that these vessels possess capillary properties and should therefore be composed of oak-leave shaped capillary LECs. To verify this assumption further, we analyzed the LEC morphology of liver lymphatics at subcellular resolution in high resolution imaged with lymphatic capillaries in the skin as reference. Albeit due to their curvature more difficult to visualize than in flat mountable organs like the mesentery, we managed to demonstrate the characteristic oak leaf shape and identified PECAM-1 and LYVE-1-rich flaps or primary valves on selected lymph vessels of the hepatic portal fields, strongly supporting the notion of lymph capillaries (Figure 4A). Further support for a capillary nature of the hepatic portal lymph vessels came from the absence of valves and a lack of SMA positive coverage.

While immune staining of sections and whole mounts revealed the localization and, more importantly, 3D architecture of lymphatics in the portal tracts, we aimed to further determine the route of fluid flow towards the initial lymph vessels. To this end, we infused primary-labeled antibodies targeting PECAM-1, LYVE-1 and LDLR in the form of a bolus injection into the portal vein (Figures 4B–D). While PECAM-1 and LYVE-1 were intended to label vascular structures, the LDLR is expressed on the basolateral membrane of hepatocytes facing the space of Disse. After 3 min of antibody circulation, ECs of dermal blood vessels in the ear were fully decorated by PECAM-1 antibodies suggesting complete systemic dissemination of the bolus. Dermal lymph vessels of the ear lacked both PECAM-1 and LYVE-1 specific staining, due to insufficient circulation time for antibodies to extravasate and reach *via* the interstitial lymph flow the dermal lymphatics. Such lymph vessel staining was observed with far longer incubation periods. In contrast, liver lymphatics in the portal tracts showed clear LYVE-1 staining (Figure 4D). Furthermore, the co-injected anti-LDLR-antibody had dominantly labelled the hepatocytes in zone 1 surrounding the hepatic sinusoids, i.e., hepatocytes located closely to the portal tracts. This staining pattern, which was pronounced in the proximity of the portal veins, suggested an immediate passage of plasma to the space of Disse where during retrograde flow to the space of Mall LDLR antibodies labelled zone 1 hepatocytes (Figures 4B,C) while upon entry into the lymph vessels LYVE-1 antibodies labelled initial capillaries (Figure 4D).

The clear spatial restriction of lymphatic vessels to the portal tracts directly raises the question which route the lymphatic vasculature takes to exit the liver. For this purpose, we examined the large portal tracts, which run close to the entrance *porta hepatis* as well as the portal vein itself to check for efferent collecting lymphatic vessels characterized by the absence of LYVE-1. Indeed, PROX-1-positive, but LYVE-1-negative vessels were present at the *porta* hinting at collecting lymphatic vessels (Figure 4E). In order to further examine the portal vein, the euthanized mouse was perfused with agarose to prevent collapsing of the vein during preparation. Analysis of the stained and optically cleared sample revealed VEGFR-3-positive, but LYVE-1-negative collecting lymph vessels with connection to intrahepatic as well as prominent extrahepatic lymphatic LYVE-1-VEGFR-3-double positive capillaries next to the portal vein (Figure 4F). Taken together, the observation of collecting vessels near the *porta* in various instances suggests a lymph flow following this hierarchical organization towards the *porta hepatis.*

### Development of lymphatic vessels in the murine liver

Developmental studies in the mouse identified cellular contributions from the foregut epithelium and the septum transversum mesenchyme to the nascent liver bud. Between E10.5 and E15.5, the fetal liver undergoes rapid expansion and becomes the major fetal hematopoietic organ. Perinatally, functional differentiation of hepatocytes and biliary epithelium commences preparing the liver for its future primarily metabolic role (Crawford et al., 2010).

**FIGURE 5:**
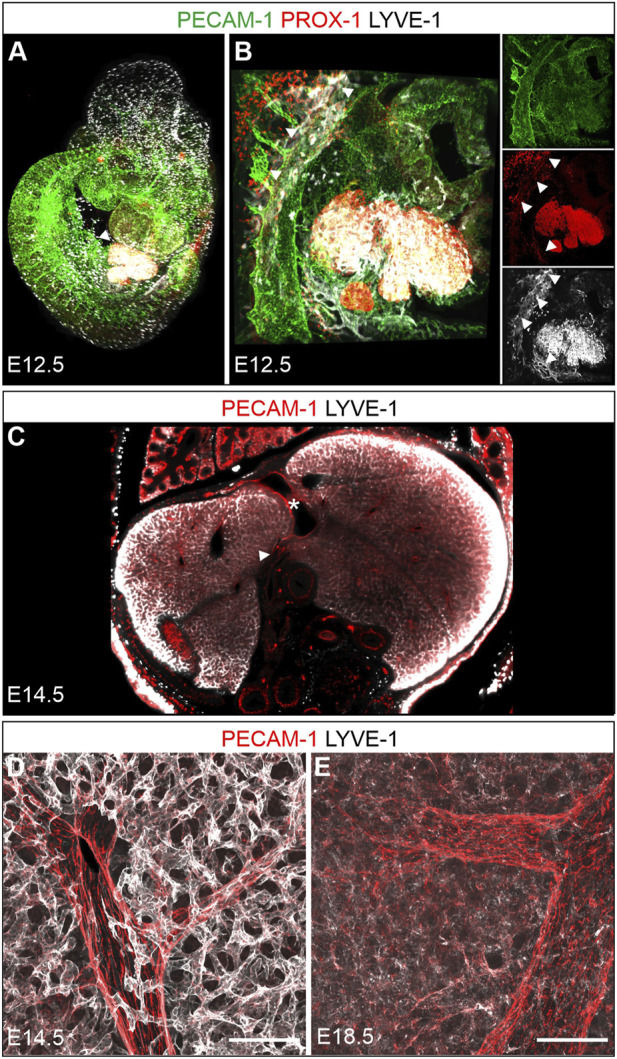
Prenatal development. A + B: 3D reconstruction of a whole mount immunostained wildtype fetus at E12.5 (overview A, developing liver B). **(A)**: The developing liver showed intense LYVE-1 and PROX-1 staining (arrowhead). **(B)**: Magnification from A, additional dorso-cranial and caudal vascular LYVE-1 and PROX-1 expression (arrowheads) indicated sites of lymph vessel formation. **(C)**: Optical coronal section through an E14.5 fetus whole mount stained for PECAM-1 and LYVE-1 revealed a homogenous network of LYVE-1 positive sinusoids with PECAM-1 most prominent on larger vessels. The heterogenous antibody penetration results in enhanced signal at the sample margins. Asterisk = ductus venosus; arrowhead = portal vein. D + E: Vibratome sections (100 µm) from livers at E14.5 and E18.5 confirmed the dense, intensely LYVE-1 positive sinusoidal meshwork at E14.5 **(D)** and demonstrated a downregulation of LYVE-1 on hepatic sinusoids at E18.5 **(E)**, identical staining conditions as in **(D)**. LYVE-1 expressing lymphatic vessels next to portal vessels were not detected at either stage. Scale bars = 100 µm.

Whole mount staining of midgestation E12.5 fetuses for PROX-1 and LYVE-1 identified besides embryonic macrophages and the known sites of lymph vessel formation also the expanding fetal liver as an organ highly expressing both proteins (Figures 5A,B). Digital rendering of a transversal optical section verified high LYVE-1 expression on the developing sinusoidal endothelium of the entire liver at E14.5 (Figure 5C). Due to the depth-dependent penetration of the antibody the LYVE-1 signal appeared stronger at the periphery of the specimen. High level LYVE-1 expression, which was apparently also not zonated, was additionally confirmed by confocal analysis of E14.5 vibratome sections (Figure 5D). Interestingly, specimens obtained from late fetuses at E18.5 and stained under identical conditions showed a strong downregulation of LYVE-1 expression, possibly indicating further hepatic differentiation (Figure 5E).

**FIGURE 6:**
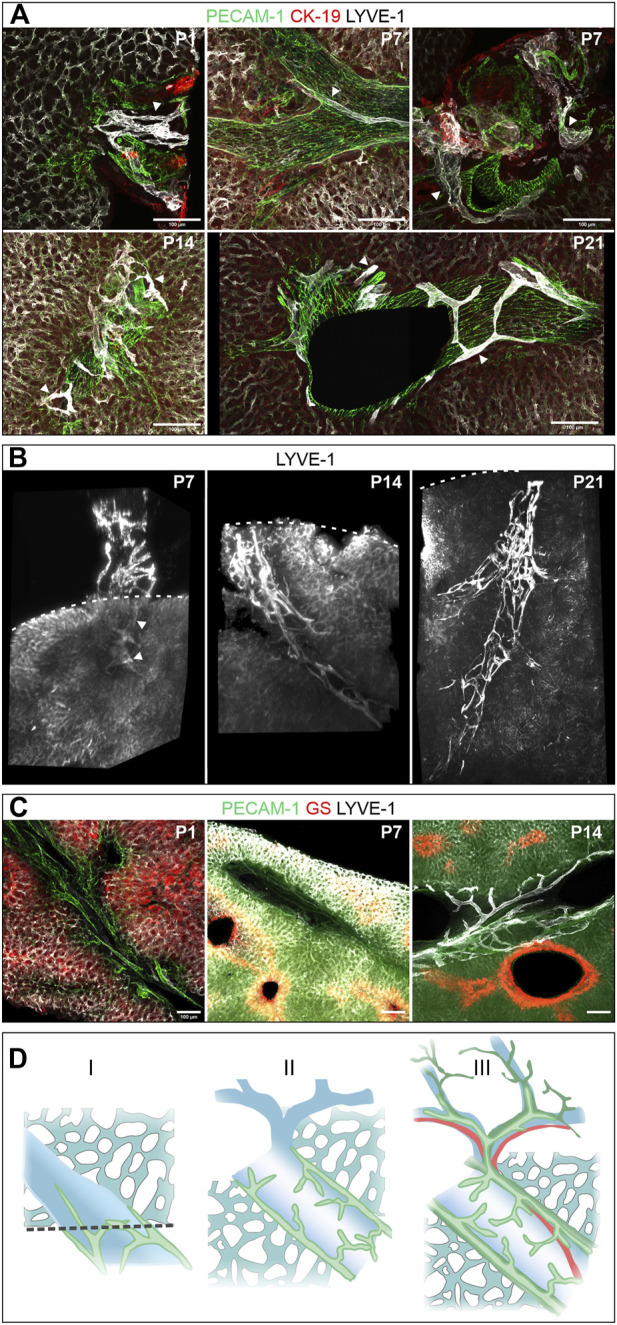
Postnatal development (the respective age is indicated in days after birth in the upper right corner of the panels). **(A)**: Vibratome sections (100 µm) of the right hepatic lobe of wildtype animals immunostained for PECAM-1, CK-19 and LYVE-1. Arrowheads denote lymphatic vessels, which were identified by high LYVE-1 expression, characteristic morphology and localization in portal tracts. Lymph vessels were restricted to large, superficial portal tracts until P7 (shown in longitudinal and cross-section), were found at increasingly deeper portal tracts at P14 und finally displayed their typical branched morphology at P21. **(B)**: 3D reconstruction of LYVE-1 stained caudate lobes showed a stepwise increase in density and expansion within the hepatic parenchyma next to a portal tract (each portal tract approximately 60–70 µm diameter). The dashed line indicates the surface of the liver lobe, arrowheads intrahepatic LYVE-1 positive lymphatic vessels at P7. **(C)**: Vibratome sections (100 µm) recapitulated the development of zonation based on the expression of the zone 3 marker GS. In contrast to the visible demarcation at P7 and P14, Zone 3 was not developed at P1. Scale bar = 100 µm. **(D)**: Scheme of the postnatal developmental stages. In the first week after birth (I), lymph vessels (green) are restricted to the proximal portal vein (blue) and intrahepatic lymph vessels are rarely found. In stage II, the lymphatic network covers large portal veins, but spare smaller branches. The full structure is established 3 weeks after birth (III) in close proximity to arteries (red) and bile ducts (dark green).

We followed the further maturation of lymphatic vessels after birth in vibratome sections of the right hepatic lobe and whole mount preparations of the caudate lobe. At P1, rather compact, LYVE-1-positive lymph vessels were found associated with large superficial portal tracts (Figure 6A, P1). This aspect was maintained until P7 (Figures 6A,B, P7). Subsequently, increasingly ramified lymph vessels were found associated with deeper portal tracts (Figures 6A,B, P14) until 3 weeks after birth the mature, fully ramified lymph vessels were found associated also with the deep portal tracts (Figures 5A,B, P21). During the same time period the initially described zonated LYVE-1 expression on sinusoidal endothelial cells and GS expression in hepatocytes was also established (Figure 5C).

In summary LYVE-1-positive lymphatic vessels appeared to form after birth with their maturation going along with the development of deep portal tracts.

### VEGF-C and VEGFR-3

**FIGURE 7:**
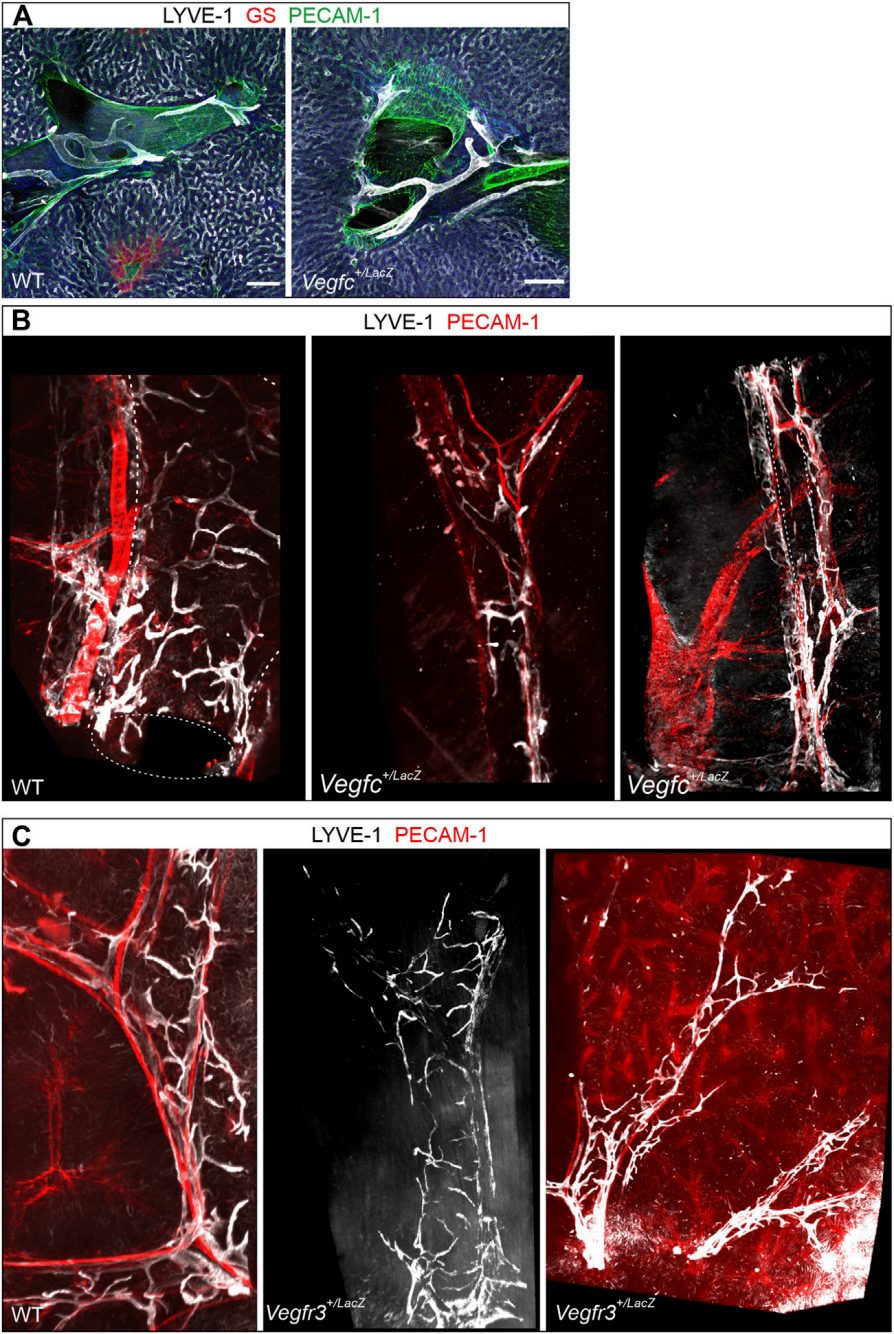
Importance of the VEGF-C/VEGFR-3 signaling axis for hepatic lymph vessel development. **(A)**: Vibratome sections (300 µm) of *Vegfc* ^*+/LacZ*^ and wildtype controls revealed no obviously discernible changes in lymphatic vessel structure at portal tracts (shown are branching points of the portal vein). Scale bar = 100 µm. **(B)**: In contrast, volumetric imaging of whole mount preparations readily identified rarified lymphatic branches clasping the portal vein and a disturbed overall spatial lymph vessel architecture. Diameter of portal tract = approximately 400 µm (first and second panel) and 200 µm (third panel). **(C)**: In contrast, in *Vegfr3* ^*+/LacZ*^ mice the architecture of perpendicularly and longitudinally oriented lymph vessel branches around the portal veins was maintained however the vessel network was more sparse. Diameter of portal tract = approximately 300 µm (first and second panel) and 150 µm (third panel).

**FIGURE 8:**
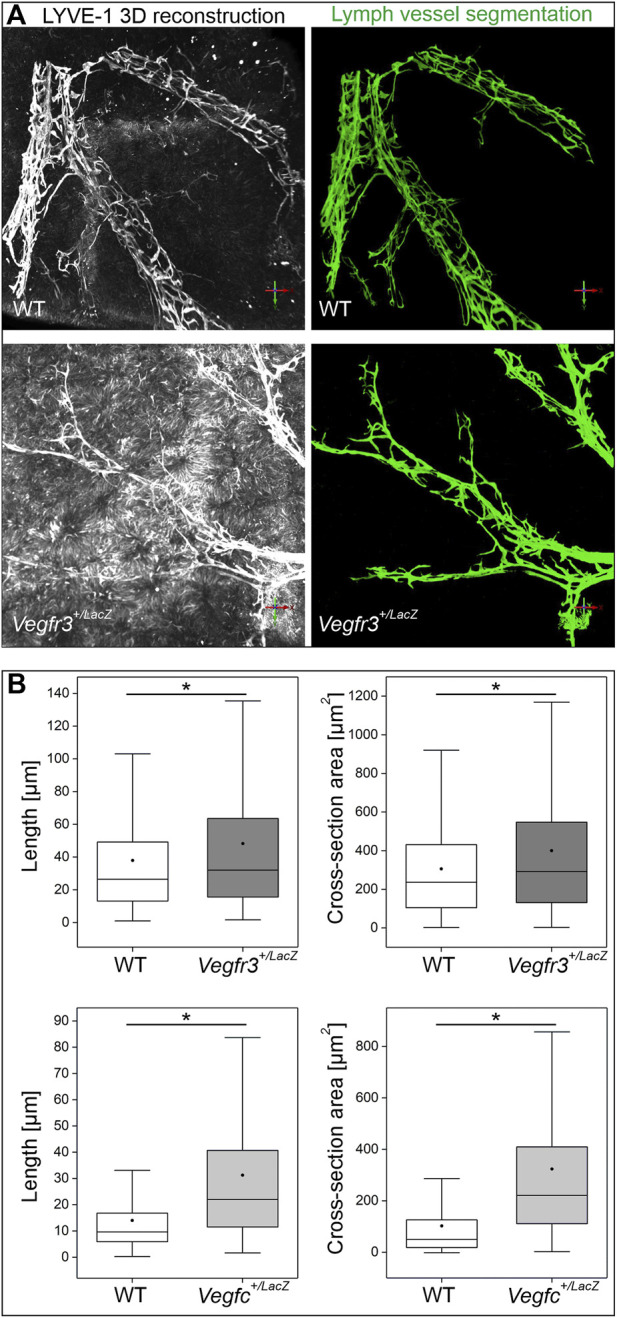
Quantification of lymphatic vascular networks in *Vegfc* ^*+/LacZ*^, *Vegfr3* ^*+/LacZ*^ and respective wildtype livers. **(A)**: Representative 3D reconstructions from LYVE-1 signal in whole mount preparations and their respective segmentation volumes after computational analysis. **(B)**: The segmentation of the lymphatic network was skeletonized and quantitative features were extracted from the underlying graph structure. *Vegfr3* ^*+/LacZ*^ and *Vegfc* ^*+/LacZ*^ livers showed significantly longer vascular segments between branching points and higher average cross-section areas. Asterisks indicate a significant result in statistical testing (Mann-Whitney-U hypothesis testing, *p* < 0.05).

Motivated by the crucial role of the VEGF-C/VEGFR-3 signaling axis for other lymphatic vessel beds forming postnatally, we probed for a potential role of this growth factor, growth factor receptor pair in hepatic lymph vessel formation. Immune staining of vibratome sections from WT and *Vegfc* ^*+/LacZ*^ livers revealed no obvious deficiencies, as branched LYVE-1-positive lymph vessels associated with the portal tracts were readily detected (Figure 7A). As the comparatively small volumes analyzed even in thick sections do not allow a full assessment of the 3D structure, we additionally analyzed whole mount immune stained liver lobes. Volumetric imaging uncovered an underestimation of morphological alterations in the section-based analysis. The full spatial representation revealed a rarefication of lymph vessel initials along entire portal tracts. Lymph vessel were less ramified and appear at places thinned out or even discontinuous, whereas the longer vascular segments in parallel to portal vessels appeared less affected (Figure 7B). When we analyzed *Vegfr3* ^*+/LacZ*^ heterozygous livers, even whole mount staining captured an intact, continuous lymphatic vascular network as apparent by simple visual inspection (Figure 7C). In order to provide a more robust evaluation and quantify changes in the lymphatic vascular morphology, we performed computational segmentation and skeletonization. Compared to their respective wildtype controls, both *Vegfr3* ^*+/LacZ*^ and *Vegfc* ^*+/LacZ*^ livers showed a significant lengthening of the initial lymphatic vessel segments between branchpoints with a higher average cross section area (Figure 8). Taken together, as expected VEGF-C and VEGFR-3 are required for the postnatal development of the full articulate hepatic lymph vessel capillaries.

## Discussion

The liver is the organ with the largest contribution to lymph production. Although lymphatic vessels are known to be important in the context of disease and tissue homeostasis, a detailed morphological description of the 3D structure of the hepatic lymph vessel bed was lacking. This study now describes the spatial arrangement of the murine liver lymphatic vasculature with cellular resolution by analyzing immunostained thick sections or whole mount preparations covering analysis of liver lobes from micro-to mesoscale.

This comprehensive approach showed that lymphatic vessels in the healthy murine liver are exclusively localized at portal tracts. Blind-ending initials were found starting near small bile ducts, covering the portal vein branches and finally growing into a tree of lymphatics including straight, larger vessels running alongside the portal vein branches and larger bile ducts. The evidence for this description is based on large multi-tile scans of vibratome sections, and staining of whole liver lobes. Light sheet microscopy allowed investigation of whole liver lobes *via* optical sectioning, which avoids tissue loss and artefacts associated with the mechanical sectioning process. Since, in general, expression of lymph vessel markers is not homogeneous throughout the entire lymphatic vasculature, we stained for several markers including LYVE-1 to identify lymph capillaries, in conjunction with VEGFR-3, which is found more widely distributed on the lymphatic vasculature. In addition, we put a particular emphasis on highlighting non-lymphatic liver structures that also expressed molecular markers found on lymphatic vessels.

The current model of lymph flow in the liver includes three routes including the portal tracts, central veins and the capsule (Tanaka and Iwakiri, 2016). While our results fully confirm the description of the portal route, we were not able to confirm lymphatic vessels underneath the hepatic capsule or near central veins. However, it is important to mention that the architecture especially of liver capsules varies between species (Ohtani and Ohtani, 2008; Rogers and Dintzis, 2018). Since the murine capsule is very thin, we are cautious to generalize our findings to species with a more pronounced liver capsule. Furthermore, our study only encompasses healthy mice, and lymphatic drainage routes may differ under pathological condition, in particular pathologies that impact on liver fluid flow like portal hypertension. Taken together, we present here a method, which, depending on the availability of suitable antibodies can be applied to specimen of all species, and will therefore allow investigation of thicker livers with capsules in the future.

Unexpectedly, LYVE-1-positive lymphatic vessels were present at the liver surface near the confluence of hepatic veins in the median. Since we found no evidence for similar structures in other lobes and the median lobe is directly facing the diaphragm, we speculate that these lymphatic vessels extend from the lymphatic network covering the diaphragm. Connections between hepatic and diaphragmatic lymphatic vessels were described earlier and until now they were classified as anastomoses emerging from the central venous lymphatics (Trutmann and Sasse, 1994b). The lymph vessels observed here could contribute to the drainage of median lobe lymph into mediastinal thoracic lymph nodes, while collectors found at the *porta hepatis* most likely transport the lymph to abdominal lymph nodes (Frenkel et al., 2020).

Immunostaining revealed a strong association of lymph vessels and bile ducts. Lymphatic vessels not only started next to small bile ducts, but also ran in very close contact to larger ducts. While it is widely accepted that the capillary plexus surrounding larger bile ducts contributes to hepatic lymph flow (Laine et al., 1979; Trutmann and Sasse, 1994b), these earliest lymphatic vessels were directly attached or even located at bile ducts too small to be equipped with a capillary plexus. We therefore speculate that these lymphatics may drain interstitial fluid originating from the bile ducts themselves rather than from surrounding vessels. This fluid could be generated upon bile modification, which includes transcellular transport of water and ions (Tabibian et al., 2013). It appears also reasonable to speculate that these lymphatic vessels might take up bile ingredients in case of leakage from the bile ducts. Similar to the enterohepatic circulation that recycles bile salts from the intestine back to the liver *via* the portal vein, the lymph flow could provide the liver with leaked bile ingredients by carrying them back to the systemic circulation. Lymphatic uptake of components destined for excretion may in this way prevent the liver from buildup of potentially toxic local deposits. This mechanism could be important in case of cholestasis upon biliary obstruction, when the pressure within the bile ducts increases. Supporting this hypothesis, bile components were found in the lymph upon biliary obstruction (Ritchie et al., 1959).

The majority of hepatic lymphatic vessels started as spiky blind ending initials and was composed of oak leaf-shaped, LYVE-1-expressing cells and thereby qualifies as lymphatic capillaries. Despite its overall composition of the ubiquitous capillary LECs the spatial structure of the hepatic lymph vessels differs from other vessel beds like the skin, diaphragm, meninges or mesentery (Petrova and Koh, 2020; Stritt et al., 2021). Since lymphatic capillaries are dedicated to the uptake of macromolecules and interstitial fluid, the large extend of hepatic lymph capillaries may explain the large production of lymph in the liver. Similar to the findings in the liver, also in other organs like the heart lymphatic capillaries represent the majority of lymphatic vessels (Brakenhielm and Alitalo, 2019).

Perinatally the liver undergoes a complex maturation accompanied by a functional switch from the major fetal hematopoietic to an adult metabolic organ. In accordance with these structural changes, we detected the formation of hepatic lymph vessels only after birth tightly associated with the formation of deep portal tracts. Not unexpected, we noted structural defects in the hepatic lymph vessels of *Vegfc* ^*+/LacZ*^ and less noticeable of *Vegfr3* ^*+/LacZ*^ mice suggesting an indispensable role of the VEGF-C/VEGFR-3 signaling axis. Similar postnatal development and dependence on VEGF-C has recently been described for meningeal lymphatic vessels (Antila et al., 2017).

We also aimed to identify fluid flow resulting in lymph generation. From antibody bolus injection into the portal vein, we suggest that plasma and molecules from the portal inflow leave the sinusoids in zone 1 and continue from the space of Disse to the space of Mall, where they enter lymphatic capillaries. Sinusoidal fenestrations with a diameter of 50–200 nm allow almost free circulation of fluid and antibodies between the vessel lumen and the space of Disse, which we visualized by detection of LDLR antibodies on the basal membrane of hepatocytes. The concentration of this LDLR staining, which was pronounced near portal tracts, indicated a preferential exit of the sinusoids proximal to the portal tracts. Since the anti-PECAM-1 antibody homogenously stained the whole sinusoidal vasculature, it is unlikely that this distribution pattern was due to insufficient access of antibodies to the sinusoids near central veins.

Collecting vessels characterized as PROX-1 or VEGFR-3-positive, but LYVE-1-negative vessels, were found on sections of the *porta* closely associated with the portal vein. Although not functionally demonstrated by our experiments, we propose that these collectors provide the outflow route for hepatic lymph. In close proximity to the *porta,* we identified lymph nodes, which should correspond to those identified as the first functional nodes filtering hepatic lymph (Barbier et al., 2012; Zheng et al., 2013; Frenkel et al., 2020).

The route for hepatic lymph generation we propose here is in agreement with the described traditional portal route (Trutmann and Sasse, 1994b; Tanaka and Iwakiri, 2016). However, our study adds functional and microanatomic evidence that lymph originates from the Disse space. Furthermore, our results argue against a bidirectional flow in the space of Disse, which was assumed to feed central venous lymphatics (Tanaka and Iwakiri, 2016). We only detected a unidirectional transport towards the space of Mall. For the transsinusoidal extravasation process, the hydrostatic transsinusoidal pressure gradient was described to act as the prominent driving force, with an osmotic gradient being negligible (Laine et al., 1979). Laine et al. (1979) invasively measured a physiological blood pressure of 7 mmHg in the PV, 2 mmHg in the inferior vena cava and 5.8 mmHg average interstitial pressure in the livers of dogs (Laine et al., 1979). The shape of the consequent porto-central sinusoidal pressure gradient likely determines the site of transsinusoidal extravasation, while the overall transsinusoidal pressure difference between the sinusoidal lumen and the space of Disse would determine the flow rate of extravasation. Observing preferential fluid extravasation near the portal vein is consistent with the measurements of Laine et al., since the higher transsinusoidal pressure gradient favors extravasation.

The liver not only metabolizes blood components, but also synthesizes large amounts of biomolecules. We have not addressed contribution of liver synthetic activity to the lymph. However, since there is a substantial fluid flow from the sinusoids towards the space of Disse and further to the space of Mall, we suggest that molecules secreted into the space of Disse by hepatocytes will exit *via* portal lymphatics rather than the bloodstream and the central veins. From this perspective hepatic lymphatic vessels would not only act as “vasa privata” in order to maintain tissue homeostasis of the liver, but in addition also act as “vasa publica” providing the body with hepatic products. This assumption is underscored by previous observations where nascent proteins were observed in hepatic lymph (Tso et al., 1983). Liver function impaired in various diseases clearly includes hepatic synthesis products needed for blood coagulation or serum albumin levels. For example, the commonly used Child Pugh score for severity of liver cirrhosis as well as the MELD score, which is needed to evaluate urgency of liver transplantations, partly rely on such surrogate parameters (Pugh et al., 1973; Malinchoc et al., 2000). Both scores are closely associated with survival rates. Our findings raise the question if lymphatic vessels may become a relevant bottle neck under pathological conditions. In case of fibrotic liver disease, disturbed lymphatic outflow may ultimately underlie the lack of hepatic enzymes. Interestingly, hepatic fibrosis was found to be associated with increased lymphangiogenesis (Yamauchi and Michitaka, 1998; Tamburini et al., 2019). While this phenomenon was interpreted as a compensatory mechanism for the release of portal pressure, it might also compensate for the deprivation in transport of hepatic products. How important this mechanism is for the whole organism and if patients at the outset of disease might benefit from facilitated lymphangiogenesis remains to be investigated.

## Data Availability

The raw data supporting the conclusions of this article will be made available by the authors, without undue reservation.
