# Supplementary material for: Volumetric imaging reveals VEGF-C-dependent formation of hepatic lymph vessels in mice
Source: Front Cell Dev Biol. 2022 Aug 16;10:949896. doi: 10.3389/fcell.2022.949896 (PMC9424489; doi:10.3389/fcell.2022.949896)
Supplement: Supplementary file 1 [file DataSheet1.pdf]

Supplementary Material

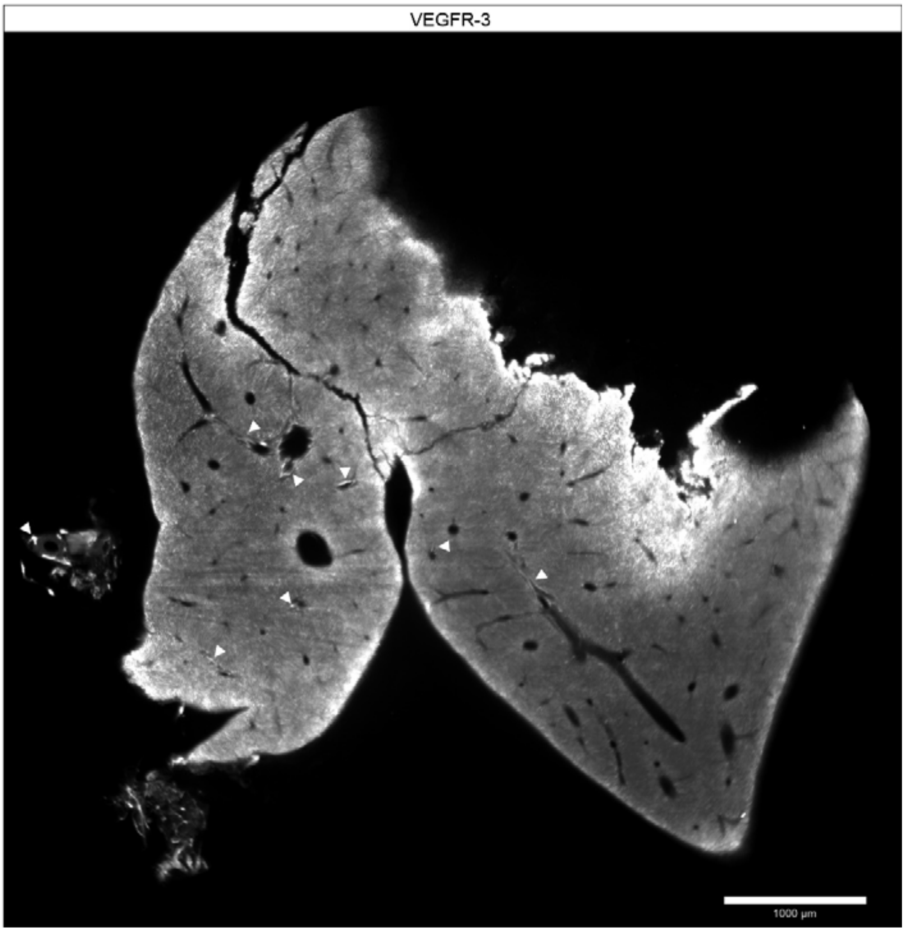

S1 Whole caudate lobe stained for VEGFR-3. Full stack (movie) of a whole mount stained caudate lobe of the murine liver and a single optical sectional plane from this specimen (image). Note the homogenous VEGFR-3 immune staining on sinusoids and the bright VEGFR-3 signal in portal tracts indicating lymph vessels (arrowheads). Scale bar = 1000 µm.

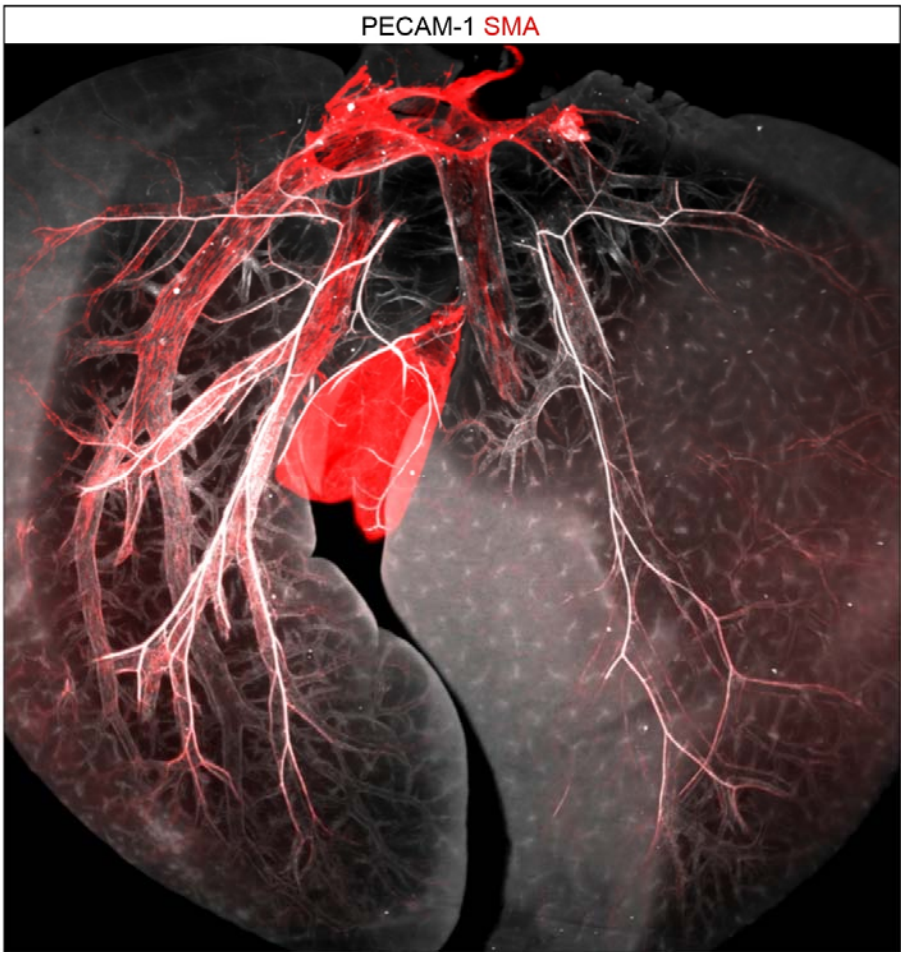

S2 Whole median lobe stained for PECAM-1 and SMA. Full stack (movie) of a whole mount stained median lobe of the murine liver and a 3D reconstruction of a sub-stack of the same specimen (image). Efferent hepatic veins and portal tracts with arteries (intense PECAM-1 and SMA signal, small diameter) and associated portal veins were identified. The gall bladder (middle) showed intense mural SMA staining.

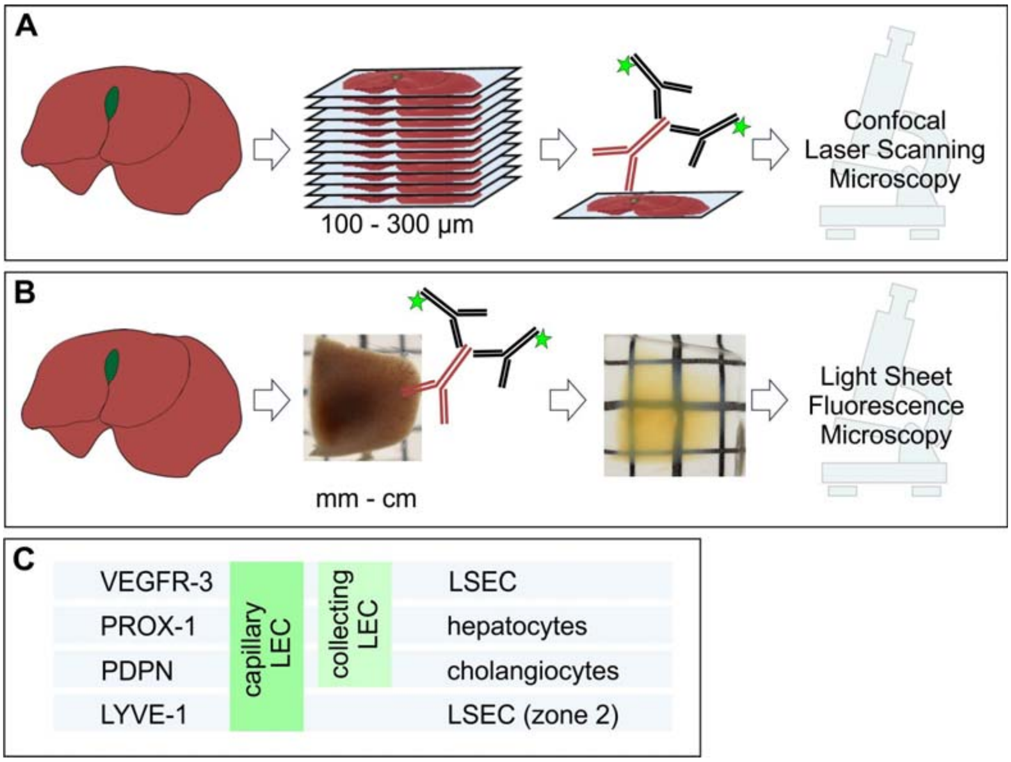

S3 Staining strategy. **A**: Thick liver sections were prepared, immunostained and analyzed using a confocal laser scanning microscope. **B**: Whole mount preparations included immunostaining of liver parts followed by clearing of the tissue for light sheet fluorescence microscopy. **C**: Lymphatic markers used for detection of capillary and collecting lymphatic endothelial cells (LEC) are also found on other hepatic structures. LSEC = liver sinusoidal endothelial cell.

Video 1: Stack of optical sections of whole caudate lobe stained for VEGFR-3

Video 2: Stack of optical sections of whole median lobe stained for PECAM-1 and SMA
